# Post-translational Regulation of GLT-1 in Neurological Diseases and Its Potential as an Effective Therapeutic Target

**DOI:** 10.3389/fnmol.2019.00164

**Published:** 2019-07-09

**Authors:** Allison R. Peterson, Devin K. Binder

**Affiliations:** Center for Glial-Neuronal Interactions, Division of Biomedical Sciences, School of Medicine, University of California, Riverside, Riverside, CA, United States

**Keywords:** GLT-1, palmitoylation, S-nitrosylation, sumoylation, ubiquitination, post-translational modifications

## Abstract

Glutamate transporter-1 (GLT-1) is a Na^+^-dependent transporter that plays a key role in glutamate homeostasis by removing excess glutamate in the central nervous system (CNS). GLT-1 dysregulation occurs in various neurological diseases including Huntington’s disease (HD), Alzheimer’s disease (AD), Parkinson’s disease (PD), amyotrophic lateral sclerosis (ALS), and epilepsy. Downregulation or dysfunction of GLT-1 has been a common finding across these diseases but how this occurs is still under investigation. This review aims to highlight post-translational regulation of GLT-1 which leads to its downregulation including sumoylation, palmitoylation, nitrosylation, ubiquitination, and subcellular localization. Various therapeutic interventions to restore GLT-1, their proposed mechanism of action and functional effects will be examined as potential treatments to attenuate the neurological symptoms associated with loss or downregulation of GLT-1.

## Introduction

Glutamate transporter-1 (GLT-1), also known as excitatory amino acid transporter 2 (EAAT2), is part of a family of Na^+^-dependent transporters that regulate extracellular glutamate homeostasis in the CNS. Na^+^-dependent glutamate transporters represent the most significant mechanism of removal of glutamate from the extracellular space and are essential in maintaining low and non-toxic concentrations of glutamate (reviewed in Danbolt, 2001). Glutamate transporter-1 is responsible for glutamate clearance from the synaptic cleft and is essential for maintaining low levels of extracellular glutamate (Levy et al., 1995). GLT-1 is expressed primarily in astrocytes and has been shown to be expressed in axon terminals of neurons (Chen et al., 2004; Furness et al., 2008; Petr et al., 2015; Zhou et al., 2019). Mice that globally lack GLT-1 develop spontaneous seizures associated with high mortality (Tanaka et al., 1997). Mice lacking GLT-1 selectively in the dorsal forebrain survive to adulthood and display transient focal seizures (Sugimoto et al., 2018). These findings revealed the important functional role of GLT-1 in extracellular glutamate modulation.

Glutamate transporter-1 expression has been shown to be altered in many neurological diseases. Huntington’s disease (HD) is an autosomal dominant disease that is characterized by degeneration of the striatum and selective cortical degeneration (Jimenez-Sanchez et al., 2017). Downregulation of GLT-1 has been observed in HD and may be responsible for the impaired glutamate uptake and glutamate toxicity observed in the R6 model of HD (Lievens et al., 2001; Estrada-Sanchez et al., 2009). Similar changes in gene expression patterns associated with transcriptional dysregulation in HD is observed in neuronal GLT-1 KO mice, suggesting neuronal GLT-1 loss in HD may lead to transcriptional dysregulation (Laprairie et al., 2019). Alzheimer’s disease (AD) is a neurodegenerative disorder which causes the gradual accumulation of Aβ and Aβ-associated proteins (Selkoe, 2000). Post-mortem tissue from individuals with a clinical history of dementia characteristic of AD show loss of GLT-1 immunoreactivity near amyloid plaques (Jacob et al., 2007). Partial loss of GLT-1 also accelerates cognitive deficit onset in the AβPPswe/PS1ΔE9 mouse model of AD (Mookherjee et al., 2011). Parkinson’s disease (PD) is a progressive neurological disorder characterized by tremor, rigidity and akinesia (Lang and Lozano, 1998). L-dopa-induced dyskinesia (LID) is often seen in patients with PD. LID severity is reduced when GLT-1 protein levels are increased in a rat 6-hydroxydopamine model of PD (Chotibut et al., 2017). Amyotrophic lateral sclerosis (ALS) is also a neurodegenerative disorder that causes both progressive and selective loss of motor neurons in the CNS (Tapia, 2014). Loss of GLT-1 has been reported during disease progression in both patients and the G85R-SOD1 mouse model of ALS (Bruijn et al., 1997; Lin et al., 1998). Epilepsy is a condition that is characterized by both recurrent and unprovoked seizures (Hauser et al., 1998). GLT-1 protein levels showed a regional decrease in epilepsy patients with hippocampal sclerosis and cortical dysplasia and in the intrahippocampal kainic acid (IHKA) model of epilepsy (Proper et al., 2002; Hubbard et al., 2016).

Glutamate transporter-1 has become an attractive target for therapeutic intervention due to its dysregulation in neurological diseases. To effectively restore GLT-1 protein levels, the mechanisms which lead to its downregulation should be investigated. Post-translational modulation of GLT-1 has been shown to contribute to its downregulation in various neurological diseases and therefore it is important to understand post-translational mechanisms to develop selective treatment strategies for individual diseases.

## Expression and Roles of Glt-1 in Neurons Vs. Astrocytes

Glutamate transporter-1 is expressed in astrocytes and presynaptic terminals of excitatory neurons. Perisynaptic astrocytic processes (PAPs) express high levels of GLT-1, with approximately 80% of total GLT-1 protein expression found in astrocytes. Neuronal GLT-1 expression accounts for only 5–10% of the total GLT-1 protein expression (Furness et al., 2008). Historically, astrocytic GLT-1 has been thought to be responsible for the majority of glutamate regulation. Interestingly, even though GLT-1 is expressed at low levels in axon terminals, studies from the Danbolt and Rosenberg labs showed that neuronal GLT-1 contributes significantly to glutamate uptake in synaptosomes (Danbolt et al., 2016; Rimmele and Rosenberg, 2016; Zhou et al., 2019). When GLT-1 is deleted in neurons, glutamate uptake capacity (V_max_) in synaptosomes is significantly reduced by approximately 40% whereas deletion of GLT-1 from astrocytes did not show a significant reduction in synaptosomal glutamate uptake capacity (V_max_). These results suggest that neuronal GLT-1 transporters, although expressed at low levels, play an important role in glutamate clearance at excitatory synapses where maintaining low levels of extracellular glutamate is critical for proper neuronal function and transmission. Importantly, however, selective ablation of GLT-1 from astrocytes results in lethal spontaneous seizures suggesting that astrocytic GLT-1 protects against fatal epilepsy (Petr et al., 2015). This finding also suggests that astrocytic GLT-1 is vital to maintain low levels of extracellular glutamate in the CNS to prevent glutamate excitotoxicity. Conversely, mice lacking neuronal GLT-1 showed normal survival and no seizures.

Differences in GLT-1 protein expression *in vivo* vs. *in vitro* is an aspect of glutamate transporter regulation that is important to take into consideration prior to exploring GLT-1 dysregulation in models of disease. Primary astrocyte cultures express mostly glutamate aspartate transporter (GLAST), another member of the Na^+^-dependent glutamate transporter family, and low levels of GLT-1 (Swanson et al., 1997; Carbone et al., 2012a). Primary astrocyte cultures also maintain a polygonal morphology, dissimilar to astrocytes of brain gray matter, and are considered undifferentiated. Interestingly, astrocytes co-cultured with neurons display a stellate morphology with branched morphology and express GLT-1 and GLAST, suggesting that neurons are responsible for regulating astrocytic glutamate transporters, particularly GLT-1. Expression of glutamate transporters has also been shown to be regulated by cAMP and neuron-conditioned medium (Swanson et al., 1997; Schlag et al., 1998). Neurons can regulate astrocytic glutamate transporters via signaling through soluble factors dependent on neuronal activity (Perego et al., 2000). The activity and cellular localization of expressed glutamate transporters can be regulated by post-translational modifications (PTMs). This review will focus on post-translational regulation of GLT-1 in neurological diseases and we will distinguish cell type-specific GLT-1 regulation if identified in the following studies (Table 1).

**TABLE 1:** Glutamate transporter-1 dysregulation in neurological disease models including potential therapeutics to prevent GLT-1 downregulation.

**Disease model**	**GLT-1 post-translational regulation**	**Potential therapeutic**	**Therapeutic effect**	**Citation**
SOD1-G93A mouse model of amyotrophic lateral sclerosis (ALS)	Caspase-3 cleaves SUMO1-conjugated GLT-1 leading to accumulation of sumoylated CTE fragments of GLT-1 in astrocyte nuclei during disease progression	Ceftriaxone	Ceftriaxone delayed loss of neurons, muscle strength and increased mouse survival	Rothstein et al., 2005; Foran et al.,2014
YAC128 mouse model of Huntington’s disease (HD)	GLT-1 uptake reduced in the striatum and cortex corresponding to a decrease in GLT-1 palmitoylation with no changes in GLT-1 protein expression	–	–	Huang et al.,2010
MPTP mouse model of Parkinson’s disease (PD)	Ubiquitin ligase Nedd4-2 mediates the PKC-dependent ubiquitination and degradation of GLT-1	Ceftriaxone	Ceftriaxone decreased behavioral deficits and neurodegeneration	Zhang et al.,2017
Lafora disease (LD) mouse model	Decreased levels of GLT-1 protein expression at the plasma membrane with no change in GLT-1 total protein expression levels suggesting a change in subcellular localization	–	–	Munoz-Ballester et al.,2016
Aβ-42 *in vitro* treatment modeling Alzheimer’s disease (AD)	Decreased levels of GLT-1 cell surface expression with no alteration in GLT-1 total protein levels in astrocyte cultures treated with Aβ-42	Vitamin E derivative (Trolox)	Normal levels of detergent-insoluble GLT-1 were restored by pretreatment with Trolox in Aβ-42-treated astrocyte cultures	Scimemi et al.,2013
R6/2 transgenic mouse model of Huntington’s disease (HD)	Decreased levels of GLT-1 expression in the cortex and striatum at 13 weeks of age when mice are severely symptomatic	Ceftriaxone	Increased cortical and striatal GLT-1 levels	Sari et al.,2010

*Glutamate transporter-1 is SUMO1-conjugated and internalized in the SOD1-G93A model of ALS. GLT-1 expression is decreased in R6/2 model of HD. GLT-1 glutamate uptake and palmitoylation is decreased in the YAC128 model of HD. GLT-1 is degraded following Nedd4-2 mediated ubiquitination in MPTP model of PD. GLT-1 is recruited to the 20S proteasome for degradation via HSP90β in the kainic acid model of TLE. GLT-1 plasma membrane protein levels are decreased in LD mice. GLT-1 cell surface expression is decreased in a model of AD.*

## Post-Translational Regulation of Glt-1

### Palmitoylation

Palmitoylation is a post-translational lipid modification that refers to the reversible thioesterification of palmitic acid to cysteine residues (Parenti et al., 1993). Palmitoylation is important for both protein function and regulation while depalmitoylation has been shown to affect protein trafficking and localization (Kong et al., 2013). Protein palmitoylation is important for neuronal development and synaptic function (el-Husseini and Bredt, 2002). Aberrant palmitoylation has been linked to neurological diseases including HD and AD (Vetrivel et al., 2009; Butland et al., 2014).

All proteins associated with the family of Na^+^-dependent glutamate transporters have been shown to be palmitoylated including GLT-1 (Kang et al., 2008). Hayden’s group at the University of British Columbia found that reduced GLT-1 palmitoylation is observed in multiple models of HD (Huang et al., 2010). GLT-1 palmitoylation was shown to be significantly reduced in the STHdh-Q111 HD striatal cell line. HD is caused by an expansion in CAG trinucleotide repeats in the gene encoding huntingtin (HTT) where increased repeat length is correlated with severity of disease (Andrew et al., 1993). The STHdh-Q111 cells express a full length mutant HTT with 111 CAG repeats. Since GLT-1 is expressed at low levels in neurons, Hayden’s group overexpressed GLT-1 in these cells to examine GLT-1 palmitoylation. [^3^H] metabolic labeling showed that the HD striatal cells had a significant reduction in GLT-1 palmitoylation compared to the wt STHdh-7Q cell line. These results suggested that there was a reduction in PTM in the presence of the mutant HTT.

Altered GLT-1 palmitoylation can impair GLT-1 glutamate uptake and may be a factor contributing to the enhancement of excitatory transmission observed in HD (Huang et al., 2010; reviewed in Andre et al., 2010). Cysteine 38 is the site of palmitoylation on GLT-1. Huang et al. generated C38s cysteine mutation of GLT-1 to abolish palmitoylation to test the functional effects of altered GLT-1 palmitoylation. A mutation in cysteine 38 eliminates palmitoylation of GLT-1 (GLT-1 C38S). COS cells expressing GLT-1 C38S showed a reduction in glutamate uptake activity compared to wt GLT-1 *in vitro* suggesting palmitoylation of GLT-1 is important for its function. Interestingly, depalmitoylation of GLT-1 was not shown to affect its subcellular localization. Inhibition of GLT-1 palmitoylation in COS cells with the palmitoylation inhibitor, 2-bromopalmitate, also reduced glutamate uptake by approximately 30% but did not affect GLT-1 cell surface expression (Huang et al., 2010).

**FIGURE 1:**
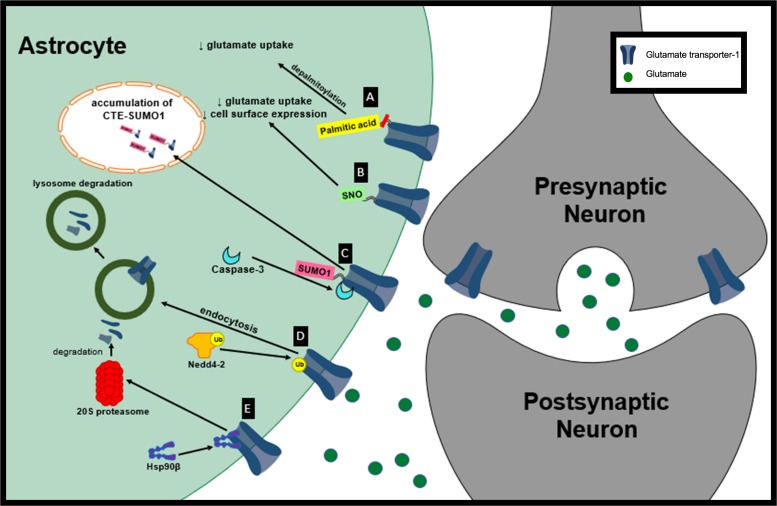
Proposed mechanisms of GLT-1 dysfunction in neurological diseases. **(A)** Depalmitoylation of GLT-1 leads to decreased glutamate uptake by GLT-1. **(B)** S-nitrosylation of GLT-1 leads to decreased glutamate uptake activity and decreased cell surface expression of GLT-1. **(C)** Sumoylated GLT-1 is cleaved by caspase-3 and its cleaved product, CTE SUMO, is internalization and accumulated in subnuclear bodies. **(D)** Nedd4-2 mediates the ubiquitination and lysosomal degradation of GLT-1. **(E)** Hsp90β recruits GLT-1 to 20S proteasome promoting GLT-1 degradation.

In a distinct model of HD, whole-brain Western blot analysis revealed that GLT-1 palmitoylation is reduced by approximately 31.8% in the brain of YAC128 mice compared with wt controls (Huang et al., 2010). YAC128 mice show reduced synaptosomal GLT-1 glutamate uptake activity in the cortex and striatum compared to wt controls measured (Table 2) (Huang et al., 2010). A selective inhibitor of GLT-1, dihydrokainate (DHK), was used to selectively determine the contribution of GLT-1 to glutamate uptake compared to GLAST. Interestingly, no change in GLT-1 protein expression was observed in the cortex, hippocampus, cerebellum or striatum in the YAC128 mouse model compared to the wt control suggesting a reduction in function not protein expression. Similar to the data observed *in vitro*, GLT-1 expression on membrane compartments in the YAC128 mice brain was not significantly different from wt controls. Together these results suggest that depalmitoylated GLT-1 has reduced function compared to palmitoylated GLT-1 (Figure 1A). Changes in GLT-1 palmitoylation can lead to reduced glutamate uptake and could contribute to the excess extracellular glutamate leading to excitotoxicity in HD (Huang et al., 2010).

**TABLE 2:** Changes in glutamate uptake in models of neurological disease.

**Post-translational modification (PTM)**	**Model**	**Experimental paradigm**	**Glutamate uptake**	**Citation**
Palmitoylation	YAC128 mouse model of PD	Synaptosomal glutamate uptake and synaptosomal DHK-sensitive glutamate uptake measured with ^3^H-labeled glutamate assay	↓Striatal synaptosomal GLT-1 glutamate uptake compared to wt ↓Cortical synaptosomal glutamate uptake compared to wt	Huang et al.,2010
Palmitoylation	COS cells expressing GLT-1 C38S	^3^H-labeled glutamate assay	↓Glutamate uptake in COS cells expressing GLT-1 C38S conpared to control	Huang et al.,2010
Nitrosylation	nNOS-/- mice	Synaptosomal glutamate uptake and synaptosomal DHK-sensitive glutamate uptake measured with ^3^H-labeled glutamate assay	↑Forebrain synaptosomal DHK-sensitive glutamate uptake mnNOS-/-conpared to wt Elimination of NO from S- nitrosocysteine ↑DHK-sensitive glutamate uptake in wt forebrain svnaDtosomes	Raju et al.,2015
Sumoylation	Primary astrocyte culture	^3^H-labeled glutamate assay	↑DHK-sensitive glutamate uptake in astrocytes overexpressing SENP1 (desumoykting enzyme) compared to control	Foran et al.,2014
Ubiquitination	Primary astrocyte culture	3H-labeled glutamate assay	↑Glutamate uptake in MPP^+^-treated astrocytes with Nedd4-2 knockdown	Zhang et al.,2017
Ubiquitination	MPTP-treated mouse model of PD	Synaptosomal glutamate uptake measured with ^3^H-labeled glutamate assay	↓Striatal synaptosome glutamate uptake compared to wt ↓Midbrain synaptosomal glutamate uptake compared to wt	Zhang et al.,2017

*YAC128 mouse model of PD shows a reduction in synaptosomal DHK-sensitive glutamate uptake in the striatum and cortex. COS cells expressing GLT-1 C38S have reduced glutamate uptake compared to wt controls. Elimination of NO in primary synaptosomes increases DHK-sensitive glutamate uptake. DHK-sensitive glutamate uptake is increased in primary astrocytes overexpressing SENP1. Knocking down Nedd4-2 in MPP+-treated astrocyte cultures increases glutamate uptake. Synaptosomal glutamate uptake is decreased in the striatum and midbrain of MPTP-treated mice.*

### S-Nitrosylation

S-nitrosylation refers to the attachment of a nitric oxide (NO) to a thiol group of a protein cysteine forming an s-nitrosothiol (SNO). S-nitrosylation plays a role in protein signaling and function (Martinez-Ruiz et al., 2011). Nitrosylation is accomplished via nitric oxide (NO) signaling during glutamatergic neurotransmission while increased S-nitrosylation of proteins has been linked to neuronal death, protein misfolding and pathogenesis in neurodegenerative diseases (Uehara et al., 2006; Cho et al., 2009; Nakamura et al., 2010). NO levels have been shown to be elevated in both epileptic children and animal models of epilepsy, which suggests NO could lead to reactive gliosis and neuronal loss seen in epileptic pathogenesis (Yasuda et al., 2001; Arhan et al., 2011). Neuronal nitric oxide synthase (nNOS) produces NO in the brain and is responsible for S-nitrosylation of proteins involved in glutamate transport.

Glutamate transporter-1 can be targeted for s-nitrosylation at Cys^373^ and Cys^562^. nNOS^–/–^ mice have an increase in DHK-sensitive glutamate uptake in forebrain synaptosomes compared to wt mice. Interestingly, nNOS^–^/^–^ mice have similar DHK-insensitive Na^+^-dependent glutamate uptake levels when compared to wt suggesting that GLT-1 independent uptake is not affected by NO (Raju et al., 2015). Raju et al. (2015) also showed that eliminating NO in wt synaptosomes by pretreatment with copper and ascorbate increased DHK-sensitive glutamate uptake in wt similar to levels observed in the nNOS^–/–^ mice. This data suggests that nitrosylated GLT-1 has a reduced synaptosomal glutamate uptake capacity (V_max_).

Exposure of human embryonic kidney (HEK)-293T cells to S-nitrosocysteine promotes S-nitrosylation *in vitro*. Interestingly, treatment of HEK-293T cells with S-nitrosocysteine did not alter GLT-1 expression but it did decrease GLT-1 surface abundance (Figure 1B). S-nitrosocysteine treatment of HEK cells reduced DHK-sensitive glutamate uptake capacity (V_max_) by 84% (Raju et al., 2015). Glutamate uptake activity is able to recover following a gradual decrease in S-nitrosylation (Raju et al., 2015). It is not known whether GLT-1 S-nitrosylation plays a role in neurological diseases but these findings introduce the opportunity for interventions to increase GLT-1 transporter activity. NO signaling is important for normal brain function but in its unregulated mode NO can cause neurotoxicity. There is evidence suggesting that NO may be involved in the pathogenesis of neurological diseases including HD, PD and AD (Boje, 2004). Even though there is currently not evidence of S-nitrosylation of GLT-1 contributing to pathogenesis in neurological diseases, nitrosylation of other proteins have been implicated in disease. For example, S-nitrosylation of peroxiredoxin 2, a neuroprotective antioxidant protein, in human PD brains inhibits is enzymatic activity and neuroprotective function (Fang et al., 2007). Glutamate uptake regulation is disrupted in many neurological diseases and GLT-1 nitrosylation could play a role in its loss of function. The nitrosylation of GLT-1 should be examined in a disease context to determine if it could be partially responsible for glutamate dysregulation.

### Sumoylation

Sumoylation is a post-translational modification that is important for various neurological functions including synaptic plasticity, neuronal excitability through ion channel regulation, and axonal transport (van Niekerk et al., 2007; Watanabe et al., 2008; Plant et al., 2011). Sumoylation involves the addition of SUMOs (small ubiquitin-like modifiers) to lysine residues via a covalent bond. Sumoylation of proteins has been shown to change their subcellular localization and expression levels (Shao et al., 2004; Fei et al., 2006).

Glutamate transporter-1 has been shown to be sumoylated under normal physiological conditions in primary astrocytes, wt rat cortex homogenates and non-ALS control post-mortem tissue with lysine 570 being the primary target for sumoylation of GLT-1 (Fei et al., 2006; Foran et al., 2014). Under oxidative stress, sumoylated GLT-1 cleaved by caspase-3 gives rise to two fragments: truncated EAAT2 and COOH terminus of EAAT2 (CTE) (Boston-Howes et al., 2006). In ALS, although sumoylation of EAAT2 is not altered, it has been hypothesized that sumoylated GLT-1 is targeted for cleavage and internalization by caspase-3 (Boston-Howes et al., 2006; Foran et al., 2014). During disease progression in the SOD1-G93A mouse model of ALS there is increased accumulation of sumoylated CTE fragments in spinal cord astrocyte nuclei (Figure 1C). Astrocytes that express CTE-SUMO1 promote motor neuron dysfunction and axonal growth abnormalities. Prolonged expression of CTE-SUMO1 is toxic to astrocytes and causes disturbances in GFAP filament organization (Foran et al., 2011).

Sumoylation of GLT-1 has been shown to dictate its intracellular localization. HEK293T cells transfected with a plasmid encoding GLT-1 express GLT-1 that is localized to the plasma membrane. HEK cells transfected with EAAT2 and a sumoylating enzyme showed an increase in the amount of GLT-1 in the cytoplasm. Similar results were observed when HEK cells were transfected to express GLT-1-SUMO1; these cells displayed considerable GLT-1 clustering inside of cells. Interestingly, primary astrocytes display a large portion of GLT-1 in the cytoplasm that is redistributed to the plasma membrane after treatment with a desumoylating enzyme, suggesting an important role of sumoylation of GLT-1 movement between cellular compartments. In primary astrocyte cultures, desumoylation of GLT-1 with SENP1 leads to an increase in DHK-sensitive glutamate uptake. Desumoylation of EAAT2 in primary astrocytes also dispersed cytoplasmic clusters of EAAT2 (Foran et al., 2014).

The Trotti group at Thomas Jefferson revealed that the extent of sumoylated EAAT2 does not change during the course of ALS progression suggesting that sumoylation is not caused by pathological mechanisms in the SOD1-G93A mouse model (Foran et al., 2014). One possibility could be that changes in caspase-3 regulation cause an increase in EAAT2 cleavage, which leads to the toxic levels of CTE-SUMO1 observed in ALS (Foran et al., 2011). A recent study showed that mutation of the caspase-3 cleavage site in astroglial GLT-1 delays disease progression and extends lifespan in the SOD1-G93A mouse model of ALS (Rosenblum et al., 2017). These results suggest that blocking the mechanisms responsible for cleavage of sumoylated GLT-1 in neurological diseases could prevent its internalization which would consequently elevate GLT-1 at the plasma membrane.

### Ubiquitination

Ubiquitination refers to the multistep enzymatic process leading to the attachment of ubiquitin to a target protein. Ubiquitinated plasma membrane proteins can be recognized and targeted for endocytic removal and degradation (Foot et al., 2017). Nedd4-2 (neuronal precursor cell expressed developmentally down-regulated 4-2) is a member of the Nedd4 family of ubiquitin ligases which catalyzes ubiquitination and degradation (Zhou et al., 2007).

Nedd4-2 has been shown to mediate the ubiquitination and internalization of GLT-1 via protein kinase C (PKC) activation (Figure 1D; Garcia-Tardon et al., 2012). Increased extracellular glutamate reduces the concentration of GLT-1 transporters on the cell surface in a dose-dependent manner in primary mixed cultures (glial cells and neurons). The mechanism responsible for the reduction in GLT-1 transporters at the plasma membrane in response to extracellular glutamate is due to the association of the adaptor protein β-arrestin with Nedd4-2 which leads to the increased ubiquitination of GLT-1 (Ibáñez et al., 2016). Elevated levels of extracellular glutamate have been observed in many neurological diseases and could potentially lead to a reduction in GLT-1 at the plasma membrane through this mechanism (Robelet et al., 2004; Soukupova et al., 2015). 1-methyl-4-phenylpyridinium (MPP^+^) is a dopaminergic neurotoxin that is used to model PD (Chun et al., 2001). Nedd4-2 interaction with GLT-1 is increased in MPP^+^-treated astrocyte cultures along with the ubiquitination of GLT-1 (Zhang et al., 2017). Ubiquitination of GLT-1 by Nedd4-2 in MPP^+^ treated astrocytes leads to its internalization and lysosomal degradation. Decreased GLT-1 expression and function in MPP^+^ treated cultures can be rescued by lysosome inhibition. Knocking down Nedd4-2, with a shRNA, decreased GLT-1 ubiquitination and increased GLT-1 expression and glutamate uptake activity in MPP+-treated astrocytes, further supporting the significance of Nedd4-2 trafficking of GLT-1 leading to its downregulation (Zhang et al., 2017).

Glutamate transporter-1 expression and glutamate uptake are decreased in the midbrain and striatum in the 1-methyl-4-phenyl-1,2,3,6-tetrahydropyridine (MPTP) mouse model of PD. GLT-1 protein expression was shown to be decreased in the midbrain and striatum in MPTP-treated mice. Synaptosomal glutamate uptake was reduced in the striatum and midbrain of MPTP-treated mice compared to controls. Interestingly, knocking down Nedd4-2 in the MPTP mouse model rescued GLT-1 protein expression and glutamate uptake (Zhang et al., 2017). Zhang et al. (2017) showed that knocking down Nedd4-2 in the MPTP mouse model of PD with a shRNA increases synaptosomal glutamate uptake. These results suggest that preventing the ubiquitination of GLT-1 could lead to reduced hyperexcitability in this model. Interestingly, other studies have shown that downregulation of Nedd4-2 leads to hyperexcitability. For example, Nedd4-2 is known to ubiquitinate voltage-gated sodium channels (Na_v_s) in dorsal root ganglion (DRG). Downregulation of Nedd4-2 leads to increased amplitudes of Na_v_s currents (Laedermann et al., 2013). Nedd4-2 heterozygous mice have increased motor activity and basal synaptic activity (Yanpallewar et al., 2016). Nedd4-2 inhibition has been shown to rescue GLT-1 protein levels, however, Nedd4-2 is responsible for the ubiquitination and regulation of numerous ion channels in the CNS suggesting that Nedd4-2 inhibition is not a good mechanism to selectively target glutamate uptake. A better approach would be to find a specific molecule that will selectively prevent Nedd4-2 and GLT-1 protein–protein interactions.

### Subcellular Localization

Glutamate transporter-1 is not homogenously expressed throughout astrocytes, rather it tends to cluster at processes and astrocytic endfeet (Schreiner et al., 2014). 67% of GLT-1 in the cortex expressed at synapses is in astrocytic processes (Melone et al., 2011).

Changes in GLT-1 subcellular localization have been documented in various neurological diseases. Primary astrocyte cultures from Lafora disease (LD, a form of intractable epilepsy) mice have reduced levels of GLT-1 at the plasma membrane but no alteration in GLT-1 total protein. These astrocytes also showed impairment in glutamate uptake capacity (Munoz-Ballester et al., 2016). Likewise, GLT-1 cell surface expression is reduced in astrocytic cultures exposed to Aβ1–42 with no alteration in GLT-1 total protein levels. Normal levels of GLT-1 can be restored in Aβ1–42 treated astrocyte cultures by pretreating with a vitamin E derivative (Scimemi et al., 2013). These findings suggest that not only reduction of glutamate transporters but GLT-1 mislocalization may play a role in neurological diseases.

## Therapeutic Strategies

### Dexamethasone

**FIGURE 2:**
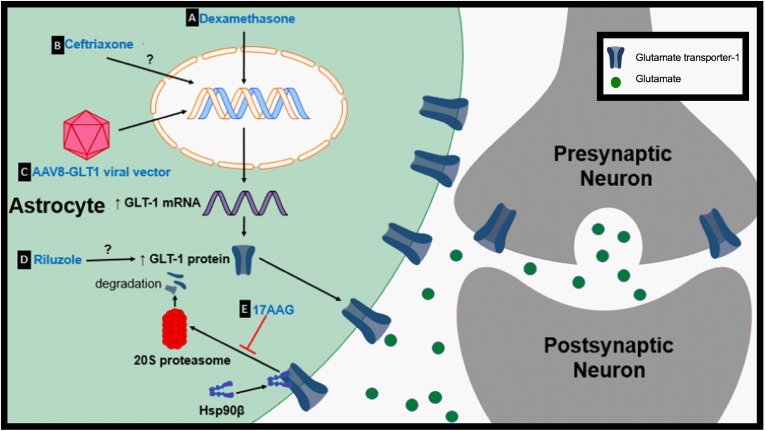
Proposed therapeutic mechanisms to prevent GLT-1 downregulation. **(A)** Dexamethasone is a transcriptional enhancer of GLT-1. **(B)** Ceftriaxone is a transcriptional activator of GLT-1. **(C)** Adeno-associated virus type 8 (AAV-8)-GLT1 vector is used to increase GLT-1 expression under the Gfa2 promoter. **(D)** The mechanism by which riluzole upregulates GLT-1 protein levels is unknown. **(E)** 17AAG prevents the degradation of GLT-1 by inhibition of Hsp90β.

Dexamethasone is a synthetic glucocorticosteroid that has been shown to be a transcriptional enhancer of GLT-1 (Figure 2A). Glucocorticoid transcriptional regulation is classically mediated through glucocorticoid receptors (GR) and receptor subtypes are expressed in primary astrocytes (Unemura et al., 2012). Dexamethasone has been reported to elevate GLT-1 transcription, protein levels and activity in cortical and striatal astrocytes *in vitro* (Zschocke et al., 2005; Carbone et al., 2012a). Dexamethasone alone was unable to increase GLT-1 protein levels in cerebellar and midbrain astrocytes but increased GLT-1 expression in cerebellar glia when used in combination with a DNA methyltransferase inhibitor (Zschocke et al., 2005). Downstream signals involved in transcription and *in vivo* models should be further explored to validate dexamethasone as a neuroprotective agent.

### Ceftriaxone

Ceftriaxone, a β-lactam antibiotic, has been shown to be increase GLT-1 expression but how ceftriaxone increases glutamate transporter expression is unclear. It is proposed that ceftriaxone elevates the transcription of GLT-1 in astrocytes through the nuclear factor-κB (NF-κB) signaling pathway, although, this is controversial due to evidence showing no change in GLT-1 protein expression with treatment in certain models (Figure 2B; Lee et al., 2008). For example, ceftriaxone treatment fails to upregulate GLT-1 or modulate glutamate uptake in striatal astrocytes following growth factor withdrawal (Fumagalli et al., 2007). It has also been hypothesized that ceftriaxone may indirectly upregulate GLT-1 through upregulation of an antioxidant defense system (LaCrosse et al., 2017). Since its discovery as a potential transcriptional activator of GLT-1, ceftriaxone’s therapeutic effects have been tested in numerous models of neurological diseases. Ceftriaxone successfully upregulated GLT-1 expression in the R6/2 model of HD when cortical and striatal GLT-1 levels are significantly reduced (Sari et al., 2010). Treatment with ceftriaxone prior to onset of epilepsy increased GLT-1 expression and decreased seizure frequency in a genetic mouse model of epilepsy (Zeng et al., 2010). Ceftriaxone increased striatal GLT-1 expression, glutamate uptake, and reduced the abnormal involuntary movements known as l-dopa-induced dyskinesia in the 6-hydroxydopamine model of PD (Chotibut et al., 2017). Ceftriaxone treatment also decreased behavioral deficits and hippocampal neurodegeneration in the MPTP model of PD (Hsu et al., 2015). Ceftriaxone delayed loss of neurons and muscle strength while increasing mouse survival in the G93A mouse model of ALS (Rothstein et al., 2005). Despite promising Stage 2 data, ceftriaxone failed to show efficacy during Stage 3 trials in ALS (Cudkowicz et al., 2014).

Treatment with ceftriaxone has been associated with negative adverse effects which might not be associated with it acting on glutamate transport suggesting that ceftriaxone affects many pathways in the CNS and is not selective to GLT-1. Ceftriaxone treatment has been shown to impair synaptic plasticity in the hippocampus and impair memory recognition (Omrani et al., 2009; Matos-Ocasio et al., 2014). Another study showed that ceftriaxone treatment results in impairment of neuronal circuits and a reduction in EEG theta power (7–9 Hz) in the frontal and parietal cortex (Bellesi et al., 2012). Critical experiments need to be performed to determine whether these adverse effects observed with ceftriaxone treatment are due to increased glutamate transporter protein expression or if they are due to ceftriaxone targeting different pathways. Ceftriaxone’s adverse effects in the CNS and mechanisms of action need to be further characterized and addressed in order to determine its potential as a modulator of glutamate uptake. If ceftriaxone does not directly target GLT-1 transcription it might not be an appropriate approach to target glutamate modulation.

### AAV8-GLT1

A direct mechanism in which genes can be delivered into cells and expressed under specific promoters can be mediated through adeno-associated virus (AAV) transduction. Recombinant AAVs have the potential to deliver normal copies of a gene that is mutated or absent to treat or prevent particular genetic diseases (Schultz and Chamberlain, 2008). Intraspinal delivery of AAV8-Gfa2-GLT1 results in an increase in astrocytic GLT-1 protein expression (Figure 2C; Li et al., 2014). Overexpression of GLT-1 through AAV8-GLT1 administration following cervical-contusion spinal cord injury (SCI) increased lesion size, phrenic nerve axonal degeneration and denervation (Li et al., 2014). Increasing GLT-1 in cervical-contusion SCI exacerbated pathology, another example of negative effects associated with modulation of glutamate transport.

However, AAV8-GLT1 injection following contusion SCI restored GLT-1 protein levels in the superficial dorsal horn and reversed heat hypersensitivity (Falnikar et al., 2016). Although AAV therapies have not been used to increase GLT-1 expression in neurological diseases, particularly diseases with GLT-1 dysfunction, gene therapy has the capability of modulating glutamate and should be further investigated. In particular, in animal model studies, AAV-induced GLT-1 upregulation can be used to isolate the effects of “pure” GLT1 upregulation from other pharmacologic effects of available drugs.

### Riluzole

Riluzole is a neuroprotective agent and is one of only two FDA-approved drug treatments for ALS. Riluzole has been shown to cause several effects including inhibition of voltage-dependent sodium channels and potassium channels (Zona et al., 1998; Urbani and Belluzzi, 2000). Riluzole has been shown to increase Na^+^-dependent glutamate uptake in synaptosomes in a dose-dependent manner but the mechanism is unknown (Figure 2D; Fumagalli et al., 2007). In striatal astrocyte cultures riluzole upregulated both GLT-1 protein levels and activity following growth factor withdrawal. 6-hydroxydopamine (6-OHDA) causes nigrostriatal degeneration and motor impairments which are characteristic of PD (Deumens et al., 2002). Riluzole treatment decreased ipsiversive rotation when challenged with amphetamine, a characteristic of 6-OHDA-treated animals that is due to loss of dopaminergic transmission, suggesting preservation of the dopaminergic system. Interestingly, riluzole did not affect GLT-1 expression in this model (Carbone et al., 2012b). The mechanisms in which riluzole modulates glutamate levels needs to be further examined to better understand its potential to rescue GLT-1 levels or function.

### HSP90 Inhibitors

Heat shock protein 90 (Hsp90) is a chaperone protein involved in proteostasis under normal physiological and pathological states (Schopf et al., 2017). Hsp90 has also been linked to protein degradation (Li et al., 2017). Hsp90 expression is increased in hippocampal formation subfields of mesial temporal lobe epilepsy (MTLE) patients compared to non-epileptic controls (Kandratavicius et al., 2014). Recent findings suggest that astrocytic Hsp90β is increased in patients with drug resistant TLE and hippocampal sclerosis (HS) (Sha et al., 2016). Astrocytic cytoplasmic Hsp90β is increased during the chronic-stage in the IHKA model of epilepsy parallel to reactive astrogliosis. Further examination indicated that specific activation of Hsp90β occurs in reactive astrocytes following an excitotoxic event. Hsp90β overexpression leads to a reduction in GLT-1 protein levels while knocking down Hsp90β increases GLT-1 expression but does not affect GLT-1 mRNA suggesting Hsp90β plays a role in regulating GLT-1 at the post-transcriptional level. The HSP90β inhibitor, 17-allylamino-17-demethoxygeldanamycin (17AAG), disrupts the interaction between Hsp90β and GLT-1 (Figures 1E, 2E). Disrupting Hsp90β’s interaction with GLT-1 prevents its recruitment and degradation by the 20S proteasome. 17AAG treatment has been shown to increase DHK-sensitive glutamate uptake in primary astrocyte cultures and hippocampal plasma membrane vesicles (PMVs) of the 17AAG treated mice, reduce epileptic seizures, and reduce astrogliosis in the IHKA model of epilepsy (Sha et al., 2016). Hsp90β is a promising new target that may be partially responsible for GLT-1 dysfunction in many neurological diseases that are accompanied by reactive astrogliosis. In addition, many different Hsp90 inhibitors are in clinical trials for cancer therapy (Jhaveri et al., 2014; Thakur et al., 2016; Cavenagh et al., 2017).

## Conclusion

Glutamate transporter-1 regulation is a critical component in homeostasis of the glutamatergic system. GLT-1 downregulation is a common occurrence seen across several neurological diseases including HD, AD, Parkinson’s disease, ALS, and epilepsy. Here, we have highlighted post-translational modifications (PTMs) that can lead to the reduction of functional GLT-1. PTMs are key mechanisms used to regulate function, localization and degradation of proteins. Under normal physiological conditions, PTMs are essential for allowing cells to respond to stimuli; but in pathogenesis, PTM dysregulation can lead to decreased function and mislocalization of proteins. In this review, we have discussed PTMs of GLT-1 in the context of neurological diseases. Selectively targeting PTMs could reveal novel therapeutics in the context of disease. For example, targeting PTMs, particularly ubiquitination, may be effective in treating particular cancers. Inhibition of the ubiquitin activating enzyme (UAE), using the small molecule inhibitor TAK-243, was shown to have anti-proliferative activity in human cancer cells (Hyer et al., 2018). Identifying changes in GLT-1 PTMs will enable modulation of both localization and function of GLT-1 in neurological diseases. Elucidation of mechanisms underlying GLT-1 dysregulation will allow design of therapeutic strategies not only to target GLT-1 at the transcriptional level but also prevent post-translational mislocalization and degradation.

## Author Contributions

AP did an exhaustive literature search and generated a complete draft of the review, and prepared the tables and both the figures. DB also reviewed the literature and provided detailed comments and edits to the review and the tables and figures.

## Conflict of Interest Statement

The authors declare that the research was conducted in the absence of any commercial or financial relationships that could be construed as a potential conflict of interest.
